# Supplementary material for: Asynchronous magnetic resonance elastography: Shear wave speed reconstruction using noise correlation of incoherent waves
Source: Magn Reson Med. 2022 Oct 27;89(3):990–1001. doi: 10.1002/mrm.29502 (PMC9792433; doi:10.1002/mrm.29502)
Supplement: Supplementary file 1 — DATA S1 MATLAB code used to generate the numerical simulations of Figure 1 [file MRM-89-990-s002.zip › k-Wave/helpfiles/example_ivp_photoacoustic_waveforms.html]

Photoacoustic Waveforms in 1D, 2D and 3D Example (k-Wave)


# Photoacoustic Waveforms in 1D, 2D and 3D Example

The time-varying pressure signals recorded from a photoacoustic source look different depending on the number of dimensions used in the simulation. This difference occurs because a point source in 1D corresponds to a plane wave in 3D, and a point source in 2D corresponds to an infinite line source in 3D. This examples shows the difference between the signals recorded in each dimension. It builds on the Simulations in One Dimension, Homogeneous Propagation Medium, and Simulations in Three Dimensions examples.

- Open the file in the MATLAB Editor
- Run the file in MATLAB

## Contents

- Waves in 1D, 2D and 3D
- Running the simulation in 1D
- Running the simulation in 2D
- Running the simulation in 3D
- Plotting the waveforms

## Waves in 1D, 2D and 3D

The fact that the characteristics of plane (1D), cylindrical (2D), and spherical (3D) wave propagation are different in some fundamental ways is often overlooked. This can lead to incorrect insight into the results from photoacoustic simulations. In particular, three key differences between 1D, 2D and 3D propagation are:

- Photoacoustic waves in 1D and 3D are compactly supported. This means they are zero outside some finite region (they "end"), whereas a waveform in 2D has an infinitely long tail. This can be understood by considering a 2D point source as an infinitely long line source in 3D. This means there will always be some signal arriving at the detector from some (increasingly distant) part of the line source. One implication for photoacoustics is that time reversal image reconstruction is not exact in 2D.
- There is no geometrical spreading in 1D, so wave amplitudes do not decay (unless there is absorption). In 2D, the waves undergo cylindrical spreading in which the acoustic energy is spread out over the growing wavefront. This means the acoustic energy is inversely proportional to radius and the acoustic pressure decays as 1/sqrt(radius). In 3D, the spreading is over a spherical wavefront, so the energy is spread over radius^2, and the pressure decays as 1/radius.
- In 1D, the shape of the initial pressure distribution will be retained in the shape of the propagating pulse. This is not true in 2D and 3D.

Note that 1D, 2D, and 3D are used here to refer to the Cartesian coordinate systems in which the variables are (x), (x, y), and (x, y, z). Other cases that could be described as 1D (such as spherically-symmetric with just a radial coordinate) or 2D (such as cylindrically-symmetric with (r, theta) as the coordinates) are not considered.

## Running the simulation in 1D

First, the common settings for all three examples are set, including the grid size, properties of the medium, size of the initial pressure distribution, source-receiver separation, time step, and length of time to run the simulation.

```
% size of the computational grid
Nx = 64;    % number of grid points in the x (row) direction
x = 1e-3;   % size of the domain in the x direction [m]
dx = x/Nx;  % grid point spacing in the x direction [m]

% define the properties of the propagation medium
medium.sound_speed = 1500;      % [m/s]

% size of the initial pressure distribution
source_radius = 2;              % [grid points]

% distance between the centre of the source and the sensor
source_sensor_distance = 10;    % [grid points]

% time array
dt = 2e-9;                      % [s]
t_end = 300e-9;                 % [s]

% computation settings
input_args = {'DataCast', 'single'};
```

The final line above sets MATLAB to run the simulations in single precision arithmetic, which is faster than double precision and more than sufficient for most simulations. The next set of commands create the k-Wave grid, the array of time points, the initial pressure distribution (source), and the sensor mask used to record the wavefield.

```
% create the computational grid
kgrid = kWaveGrid(Nx, dx);

% create the time array
kgrid.setTime(round(t_end / dt) + 1, dt);

% create initial pressure distribution
source.p0 = zeros(Nx, 1);
source.p0(Nx/2 - source_radius:Nx/2 + source_radius) = 1;

% define a single sensor point
sensor.mask = zeros(Nx, 1);
sensor.mask(Nx/2 + source_sensor_distance) = 1;	
```

Finally, the 1D example is run.

```
% run the simulation
sensor_data_1D = kspaceFirstOrder1D(kgrid, medium, source, sensor, input_args{:});
```

## Running the simulation in 2D

Running the simulation in 2D is very similar, except the initial pressure distribution is defined as a disc (filled circle) using `makeDisc`, and the sensor mask is defined as a single pixel in two-dimensions.

```
% create the computational grid
kgrid = kWaveGrid(Nx, dx, Nx, dx);

% create initial pressure distribution
source.p0 = makeDisc(Nx, Nx, Nx/2, Nx/2, source_radius);

% define a single sensor point
sensor.mask = zeros(Nx, Nx);
sensor.mask(Nx/2 - source_sensor_distance, Nx/2) = 1;

% run the simulation
sensor_data_2D = kspaceFirstOrder2D(kgrid, medium, source, sensor, input_args{:});
```

## Running the simulation in 3D

The 3D example follows the same pattern, except now the source is defined as a ball (filled sphere) using `makeBall`.

```
% create the computational grid
kgrid = kWaveGrid(Nx, dx, Nx, dx, Nx, dx);

% create initial pressure distribution
source.p0 = makeBall(Nx, Nx, Nx, Nx/2, Nx/2, Nx/2, source_radius);

% define a single sensor point
sensor.mask = zeros(Nx, Nx, Nx);
sensor.mask(Nx/2 - source_sensor_distance, Nx/2, Nx/2) = 1;

% run the simulation
sensor_data_3D = kspaceFirstOrder3D(kgrid, medium, source, sensor, input_args{:});
```

## Plotting the waveforms

The three recorded time series for 1D, 2D and 3D are shown below (the magnitudes have been normalised). The fact that the 1D and 3D waveforms are compactly supported can be clearly seen.
